# Domestic Cats (*Felis silvestris catus*) Do Not Show Signs of Secure Attachment to Their Owners

**DOI:** 10.1371/journal.pone.0135109

**Published:** 2015-09-02

**Authors:** Alice Potter, Daniel Simon Mills

**Affiliations:** Animal Behaviour Cognition and Welfare Group, School of Life Sciences, University of Lincoln, Lincoln, Lincolnshire, United Kingdom; Birkbeck, University of London, UNITED KINGDOM

## Abstract

The Ainsworth Strange Situation Test (SST) has been widely used to demonstrate that the bond between both children and dogs to their primary carer typically meets the requirements of a secure attachment (i.e. the carer being perceived as a focus of safety and security in otherwise threatening environments), and has been adapted for cats with a similar claim made. However methodological problems in this latter research make the claim that the cat-owner bond is typically a secure attachment, operationally definable by its behaviour in the SST, questionable. We therefore developed an adapted version of the SST with the necessary methodological controls which include a full counterbalance of the procedure. A cross-over design experiment with 20 cat-owner pairs (10 each undertaking one of the two versions of the SST first) and continuous focal sampling was used to record the duration of a range of behavioural states expressed by the cats that might be useful for assessing secure attachment. Since data were not normally distributed, non-parametric analyses were used on those behaviours shown to be reliable across the two versions of the test (which excluded much cat behaviour). Although cats vocalised more when the owner rather the stranger left the cat with the other individual, there was no other evidence consistent with the interpretation of the bond between a cat and its owner meeting the requirements of a secure attachment. These results are consistent with the view that adult cats are typically quite autonomous, even in their social relationships, and not necessarily dependent on others to provide a sense of security and safety. It is concluded that alternative methods need to be developed to characterise the normal psychological features of the cat-owner bond.

## Introduction

The domestic cat (*Felis silvestris catus*) has recently passed the dog as the most popular companion animal within Europe [1], [2]. Ease of care, ability to live in a small residence and the capacity to cope with being left alone for long periods of time have been reported as reasons for this popularity [1], [3], [4], [5]. Indeed, some have suggested that cats are ‘ideal’ companions for owners who work long hours [5]. However, there is evidence to indicate that some cats may show signs of separation distress in the absence of their owner [6] and it has been suggested that the cat-owner bond may be a form of attachment similar to that which exists between a dog or child and its primary carer [7]. Bowlby [8] [9] described attachment as an enduring psychological bond, that serves to improve an infant’s chances of survival by keeping it close to its mother. In this context the term “attachment” has a precise operational definition relating to the provision of safety and security, and is not simply an affectionate bond; it has several objectively definable characteristics: attached individuals seek to maintain proximity and contact with the attachment figure, attached individuals become distressed when involuntarily separated and show signs of pleasure upon their return, attachment figures act as a safe haven to which the attached individual will return when frightened by the environment, attachment figures act as a secure-base from which the attached individual can move off and engage confidently in activities [10]. None of these alone is sufficient to demonstrate or infer secure attachment, but many of these features are assessed within the Strange Situation Test (SST) developed by Ainsworth [11] for this purpose. The procedure involves placing a subject in an unfamiliar room (strange situation–to provoke a sense of insecurity) together with its carer (potential attachment figure) and a stranger (social control to which there should be no attachment) followed by a series of episodes of separation from, and reunions with, their carer and the stranger. The normal healthy response in this context involves a differentiation between the carer and the stranger in the support they provide to the subject in this challenging environment. This can be used to imply the type of attachment that exists between the subject and care. In this way a secure attachment style can be operationally defined; other styles of response are considered problematic [11]. The test was originally developed to investigate mother–infant attachment, but has been used and adapted for studying attachment between other species and their carers e.g. chimpanzees [12], dogs [13],[14], [15], [16], [17], [18], [19], [20] and hand-reared wolves [17]. Its application to dogs was inspired by the resemblance of the dog-owner bond to that which exists between a child and parent [13], [14] and there is now strong evidence to indicate that the typical dog-human bond also includes the requirements of a secure attachment defined in this way [18]. The well-developed sociality of dogs may be particularly important in this regard [14]. Nonetheless both cats and dogs appear to show separation related problems, which it has been suggested might be associated with attachment to owners [21] and it is increasingly recognised that cats (*Felis sylvestris catus*) are perhaps more social than traditionally thought even with their own species [22], [23], [24], [25], [26], [27], [28], [29]. Co-operative colonies of related females arising as a result of the availability of key resources are well documented [24], [25], but even male cats, especially those neutered, are known to be social [28]. Preferred associates may be identified from affiliative behaviours such as allorubbing and allogrooming [22] and this type of activity may be used to assess the social bond that exists between the individuals involved [26]. It is therefore clear that cats have the capacity to form social intraspecific relationships, and this may underpin the form of relationship they form with humans, especially those with whom they share a home.

The extent to which cats demonstrate sociality towards humans appears to be influenced by a wide range of factors [30], [31]. Kittens are reported to have a sensitive phase of socialisation towards humans between their second and seventh week of life [32]. During this time exposure to humans, amount of handling, number of handlers and presence of the queen have all been found to influence sociality towards humans [32], [33]. A range of human-related factors also influence the development of the social behaviour expressed by cats towards people [34], [35]. Given these findings and the tendency of owners to consider pet cats as part of the family [36], it seems reasonable to examine whether the typical bond shown by pet cats *towards their owners* also involves a form of secure attachment that provides additional safety and security. Using a modified version of the Ainsworth SST, Edwards *et al*., [7] have claimed, on the basis of a preliminary study, that this is indeed the case. They reported that cats only played in the presence of their owner, vocalised more when left alone, engaged in more locomotion/exploration while the owner was present and were more alert in the presence of the stranger [7]. However their conclusion that their use of a modified SST demonstrates that the cat-owner bond typically meets the requirements of a secure attachment is questionable on several grounds, due to methodological flaws in their study, which might account for the differences observed. Firstly, the experience of the cat within the procedure in relation to the owner and stranger was not equivalent. For example when analysing the cats’ behaviour towards the owners, Edwards *et al*., [7] use data from two episodes within the test, neither of which follow an episode of the cat being alone, whereas the assessment of the cats’ behaviour towards the strangers depends on only one episode which follows an episode of isolation. The different conditions applied to the cats in the time preceding the episode when it is alone with the owner and stranger, may therefore explain differences in the cats’ behaviour towards these individuals rather than the relationship the cat has with each of them. Secondly, Edwards *et al*., [7] also fail to control for a possible episode-order effect. Episode-order describes the sequence in which the owner and stranger participate in the procedure and this has the potential to affect the cat’s behaviour, as the cat may alter its behaviour in relation to the strange situation over time, regardless of who is present. The use of a counterbalanced procedure in which the sequence is reversed for half of the subjects can control for this potential confound [18]. Thirdly, Edwards *et al*., [7] did not analyse the data from all episodes, but only four of them (episodes 4–7). This meant their data came from 6 minutes of observation of the cat with the owner, 3 minutes with the stranger and 3 minutes when the cat was alone. This again confounds the ability to ascribe differences in behaviour towards the two types of human subject involved in the test to the relationship, rather than to the methodological features of the study. Finally, it is also assumed by Edwards *et al*., [7] that the cat behaviour observed is reliable, (i.e. that a given cat would consistently show this type of response in this type of situation); given the large number of variables assessed, spurious findings due to Type I statistical errors are a risk. Therefore the study reported here re-examined the issue of secure attachment by cats to their carers, in a way that addresses these concerns by using a cross over design experiment with an improved and counterbalanced modification of the Ainsworth SST. Our first aim was to assess the robustness of potential measures of cat attachment within the SST; our second aim was to assess whether those behaviours found to be suitably robust and relevant indicate that the cat-owner relationship meets the requirements of a secure attachment as defined within the SST.

## Materials and Methods

### Ethics statement

As part of the protocol for this study, all owners provided written informed consent to their and their cat’s participation. Owners were allowed to withdraw this consent at any time without giving reason, and no data from these subjects would be used. The full protocol, including consent procedures, was approved by the University of Lincoln School of Life Sciences Research Ethics Committee, after specific consideration of the ethical factors relating to both the humans (owners- EA2) and non-human animals (cats- EA3) involved.

### Subjects and Participants

A convenience sample of twenty owner-cat dyads was recruited through personal contact and advertisements in local pet related businesses. All participants lived within 5 miles (8km) of the test area. Owners were all adults, with four males and 16 females agreeing to take part. A broad spectrum of ownership lifestyles were represented, with ten of the owners in full-time employment, 3 in part-time employment, 3 students and 2 unemployed. The cat subjects were 13 males and 7 females whose ages ranged between 1–9 years old (mean age ± SD; 5.05 years ± 3.17). One female cat and all males had been neutered, and no entire female was in season. Two cats were pedigree British Shorthairs and the remaining 18 subjects were Domestic shorthairs (15) and Domestic longhairs (3). All cats had been in their current home for a minimum of ten months. Eleven of the cats lived with at least one other cat (range 1–4) and two lived with a dog in the home. Nineteen of the cats had regular access to the outside. All cats were free from either overt or known on-going medical conditions. All but one owner provided information relating to the experience of the cats of strange situations. Only two had much experience of strange situations away from the current owner, since being taken in by the current owner (having lived away from the owner for a period of time), eight were known to have moved home with the owner. All except one of the cats were used to being transported in a cat carrier.

### Testing area and materials

**Fig 1:**
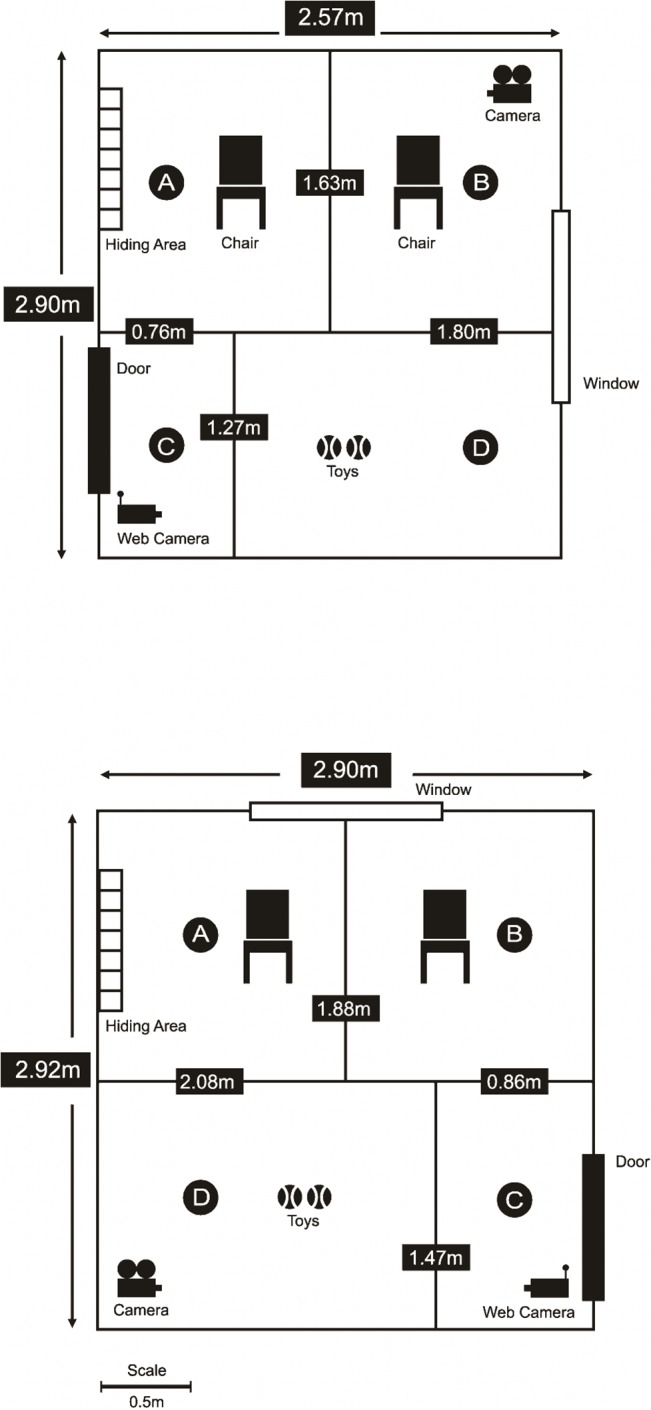
Graphical illustration of layout and dimensions of testing rooms (1) and (2), Each room was divided into four areas A) owner/stranger, B) owner/stranger, C) door, D) play.

Two similar, plain rooms were used for the study (Fig 1), in order to ensure an equivalent strange physical environment in each test. Test rooms were unfamiliar to all cat subjects. Both rooms were equipped with two chairs (for the owner and stranger), three cat toys (two balls and a string and rod toy) and a small area (approx. 80cm by 75cm by 35cm, with a partially occluded entrance) in which cats could hide. Windows in both rooms were covered to avoid any visual distraction from outside. Within both rooms a video camera (Flip Video Ultra HD) mounted on a tripod was set up to record the test period and a web camera connected through a live feed to a monitor located outside the test room was mounted above each doorway. The web camera allowed both the experimenter to follow the procedure and the owners to observe their cat during episodes in which they were not present. The rooms were divided by strips of white tape into four zones: i) region around owner’s chair, ii) region around stranger’s chair, iii) door, iv) play area (Fig 1). In order to control for any effects of spatial location of owner, the owners were pseudo-randomly assigned to one of the two chairs available (Fig 1) in the first test and then allocated the other chair in the repeat test. The test rooms and all equipment were thoroughly cleaned with an enzymatic cleaner (*Urine-Off for Cats and Kittens*) before and after each test to remove cat related odours, between tests.

### Procedure

The procedure comprised of two conditions: modified (A) and reversed modified (B) version of the Ainsworth SST both consisting of nine 3 minute episodes in which the cat is either alone or with the owner and/or a stranger in order to assess how it responds to a series of procedures designed to alter the level and form of social support available to it, or trigger seeking out of an attachment figure (see Table 1 for details of the procedures and “Data collection and analysis plan” below for details of the specific predictions made in different circumstances if a cat is securely attached to its owner). Hereafter the convention of a letter followed by a number is used to refer to conditions and episodes as described in Table 1. Hence, A2 refers to condition A episode 2. The Ainsworth SST was extended from six to nine episodes in order to allow the owner and stranger to take part in an equal number of episodes, separations and reunions with the cat subjects. As with the original Ainsworth procedure the duration of each episode was three minutes. In condition B the episode-order of condition A was reversed. This reversal of episode sequence balanced the order in which the owner and stranger participated in the procedure across the two tests (Table 1).

**Table 1:** Outline of episode sequence and protocol for the modified (A) and reversed (B) Strange Situation Test conditions. The following effects across the episodes would be consistent with secure attachment towards the carer over the social control (stranger); increased passive behaviour, exploration and social play in the presence of the carer; greater seeking of proximity and attempts to maintain proximity/contact with the carer; greater vocalisation when separated from the carer; increased vigilance and orientation to the door when the carer is absent.

Episode No	Modified (A)	Reversed (B)	Protocol	Time (minutes)
1	Owner, stranger and cat	Owner, stranger and cat	No interaction between owner, stranger and cat unless initiated by the cat for first minute. Conversation between owner and stranger for second minute. Third minute owner/stranger initiates play with cat. At the end of the 3 minutes that individual leaves the room unobtrusively.	3
2	Stranger and cat *1st separation with owner	Owner and cat *1 ^*st*^ *separation with stranger*	Owner/Stranger continues to play with cat for first minute and then provides attention only if the cat seeks it. Owner/Stranger leaves the room at the end of 3 minutes.	3
3	Cat alone *1 ^*st*^ *separation with stranger*	Cat alone *1 ^*st*^ *separation with owner*	Cat alone.	3
4	Owner and cat *1 ^*st*^ *reunion with owner*	Stranger and cat *1 ^*st*^ *reunion with stranger*	Owner/Stranger enters room and pauses at the door once inside to allow the cat to greet. Owner/Stranger plays with cat for first minute and then only provides attention if the cat seeks it.	3
5	Owner, stranger and cat *1 ^*st*^ *reunion with stranger*	Owner, stranger and cat *1 ^*st*^ *reunion with owner*	Owner/Stranger enters room and pauses at door once inside to allow cat to greet. No interaction from owner or stranger unless initiated by the cat for the first minute. Conversation between owner and stranger during the second minute. In the third minute owner/stranger initiates play with the cat. At the end of the third minute owner/stranger leaves the room unobtrusively.	3
6	Owner and cat*2 ^*nd*^ *separation with stranger*	Stranger and cat *2 ^*nd*^ *separation with owner*	Owner/Stranger continues to play with cat for first minute and then provides attention only if the cat seeks it. Owner/Stranger leaves the room at the end of the third minute.	3
7	Cat alone*2 ^*nd*^ *separation with owner*	Cat alone*2 ^*nd*^ *separation with stranger*	Cat alone.	3
8	Stranger and cat *2 ^*nd*^ *reunion with stranger*	Owner and cat*2 ^*nd*^ *reunion with owner*	Owner/Stranger enters room and pauses at door once inside to allow cat to greet. Owner/Stranger plays with cat for first minute and then only provides attention if the cat seeks it.	3
9	Owner, stranger and cat *2 ^*nd*^ *reunion with owner*	Owner, stranger and cat *2 ^*nd*^ *reunion with stranger*	Owner/Stranger enters the room and pauses at the door once inside to allow cat to greet. No interaction between owner, stranger and cat unless initiated by the cat.	3

All owner-cat pairs participated in both conditions (A and B) in different rooms set up for the procedure, and were tested within 5 minutes of arrival at the test site. Ten subjects were pseudo-randomly assigned to group 1 (condition A followed by condition B) and the other ten to group 2 (condition B followed by condition A). For all subjects a period of at least two weeks elapsed between participation in the two conditions. Two females of similar height, build and appearance were used as the stranger (one for each condition). Testing was conducted over a seven week period between May and July 2012. To accommodate the schedules of subjects the tests were conducted at a variety of times between 09:30 and 19:00 Monday to Sunday. On average tests on the same subject were conducted within an hour of the same time of day on the two occasions (mean difference 51.75 mins, mode = 0).

### Data collection and analysis plan

Following a pilot study and review of previous research [7], a list of behaviours was drawn up for recording (See S1 File). Continuous focal sampling of video recordings of the cat’s behaviour was undertaken using Solomon Coder (Beta 12.07.10) to record the duration of these behaviours [37] during each episode of each condition. In order to minimise the subsequent risk of error due to multiple statistical testing, some functionally related behavioural categories were grouped. Specifically ‘passive exploration’, ‘active exploration’ and ‘locomotion’ were grouped into ‘exploration/locomotion’ and ‘approaching/orientation to a person’ and ‘following’ were grouped into ‘proximate owner/stranger’. Behavioural measures were then classified *a priori* according to their putative relationship to one or more of the operationally definable characteristics of attachment, and only these measures considered for statistical analysis (Table 2). Three characteristics of attachment (proximity/contact seeking, secure-base effect and distress due to separation) could be assessed. Firstly, the owner should be a preferred social companion to the stranger as evidenced by the cat seeking proximity and attempting to maintain proximity/contact more with the owner than with the stranger. Secondly, if the owner acts as a secure base, it was predicted that there should be more passive behaviour, exploration and social play in the presence of the owner compared to the presence of the stranger. Thirdly, cats should be more distressed by the absence of the owner than the stranger, and so it was predicted that cats should vocalise more when separated from the owner compared to the stranger, and show greater vigilance and orientation to the door when the owner is absent compared to the when the stranger is absent. No single measure would be sufficient to conclude that the relationship between the cat and its owner is a secure attachment, rather the evidence from all of these tests would need to be considered overall.

**Table 2:** List of behaviours statistically analysed and the corresponding characteristic of attachment they may indicate. See S1 File for definition of the specific behaviours as used for their identification from the video.

Characteristic of attachment	Behaviour
Proximity/contact seeking:	Proximate owner/stranger inc. following and approach
	Physical contact owner/stranger
	Marking owner/stranger
Secure-base effect:	Exploration/locomotion
	Passive behaviours
	Social play owner/stranger
Distress when separated:	Vocalise
	Approaching/orientating to the door
	Vigilance

Any cat subjects who remained in the hiding place throughout the experimental testing procedures were removed from the data analysis, since they provided no useful data.

All statistical analysis was conducted in Minitab 16 (Minitab Ltd). Normality was assessed using a Kolmogorov-Smirnov test and since data were not normally distributed a non-parametric analysis was undertaken, in accordance with the procedure described by Jones and Kenward (2003) [38] for the analysis of cross over design experiments.

#### Assessment of the reliability of the behaviour of cats in comparable situations

In the first instance it was important to establish the reliability of the behaviour that had the potential to be used to assess attachment by cats. The counterbalancing of the procedure and within subjects design of the experiment, meant that it was possible to systematically analyse the data to detect significant differences in the behaviour within subjects in the same circumstances but at different times. This allowed a rigorous assessment of the robustness of each potential measure, using the following process. Comparable measures of behaviour based on either single episodes (e.g. vocalising during A2 versus B6) or combinations of episode (e.g. vocalising in the two episodes A2 and A8 versus B6 and B4) were identified (see S2 File for a full list). Next, in accordance with Jones and Kenward (2003) [38], the occurrence of a significant interaction between the order in which subjects were tested (test-order) and the condition on a given behaviour was examined first using a Mann-Whitney test. Only if there was no significant interaction could the behaviour be taken forward for further consideration as a potentially useful measure. The next stage of analysis examined if there was a significant test-order effect on the given behaviour. If a significant test-order effect was found then that behaviour could not be used in any analysis of attachment between the two conditions (A versus B). Next, because each social situation was replicated within a condition, the significance of any episode-order effect on the behaviour was examined (i.e. the effect of time within a test). If a significant episode-order effect was found then that behaviour could not be used in any analysis of the behaviour within episodes occurring at a different time but within the same condition. If the behaviour was affected by both test-order effect and episode order then it could not be used as a potential measure to assess attachment. Only behaviour measures that did not meet any of these exclusion criteria were robust enough to assess whether the cats showed signs of attachment in the modified SST.

Individuals who hid during an entire condition were excluded from analytical consideration.

#### Assessment of attachment within the modified SST

Following assessment of the reliability of the potential behaviour measures, those behaviours that were deemed suitably robust were used to determine if significant differences occurred within-subjects that were consistent with signs of secure attachment of the cat to its owner, using Wilcoxon’s signed rank tests. Specifically, the predictions outlined in the “Data collection and analysis plan” (above) were tested where there were suitable data.

## Results

**Fig 2:**
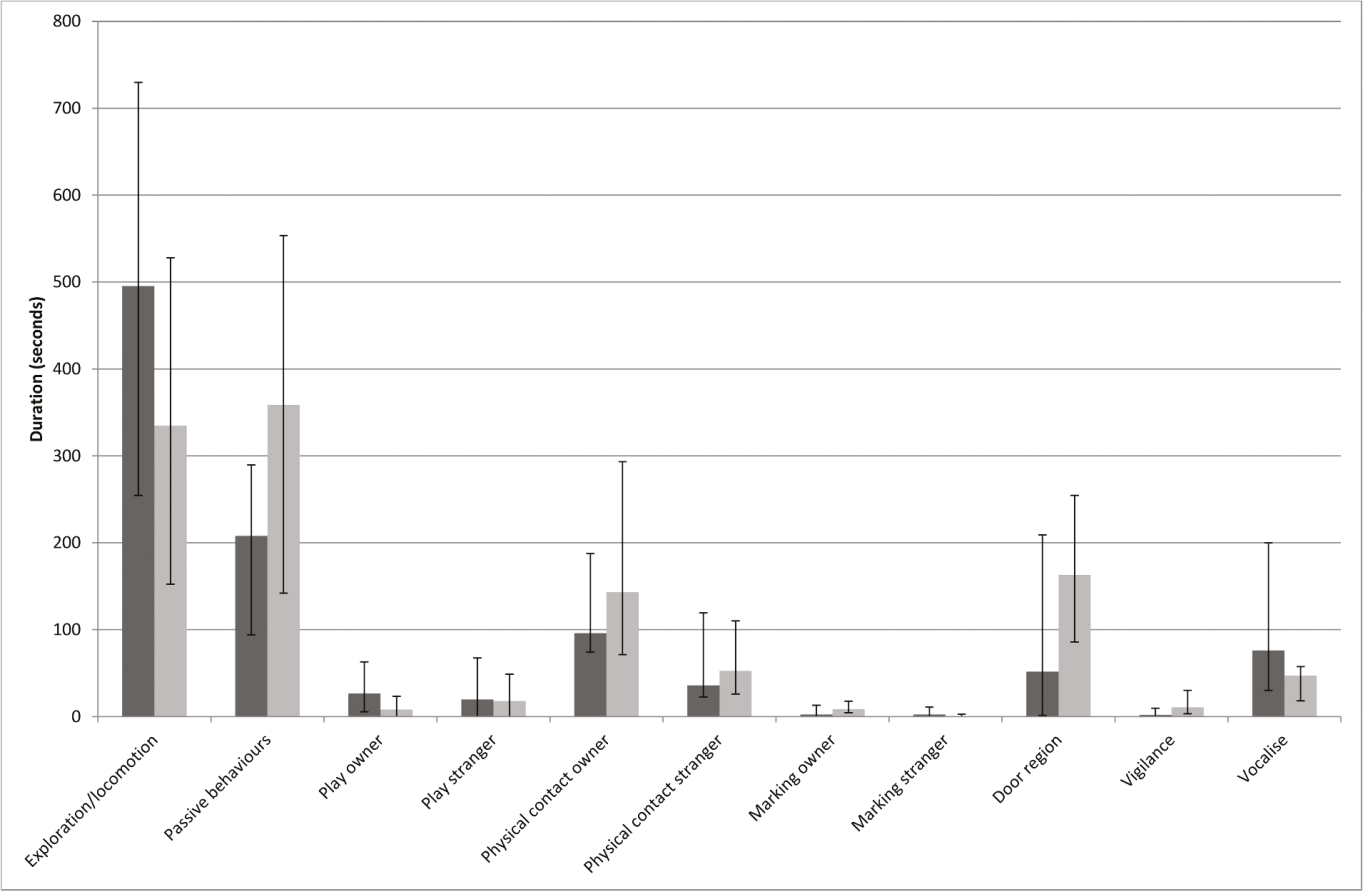
Median time (and interquartile range) in seconds, spent in various activities by cats in the two conditions. Darker bar refers to condition A and lighter bar to condition B.

Two cat subjects (1 male neutered, 6 years old, 1 female neutered 2 years old) hid during an entire experimental testing period and were therefore removed from the data analysis. None were removed due to concerns over their welfare during the test. This left data from 18 subjects for analysis. The median duration and interquartile range of the behaviours of interest in the two conditions are shown in Fig 2. This appears to show widespread inconsistency in the cat’s behaviour across the two conditions. This highlights the need to assess the scientific reliability of these behaviours, since behaviours that are inconsistent cannot be used to reliably assess supposedly stable traits such as attachment.

Full results from the assessment of behavioural reliability can be found in the S3 File, but in the following sections we consider further the value of the various behaviours that could potentially contribute to the assessment of different facets of attachment, starting with their robustness as potential psychometric measures. To be suitable, measures of the same situation should show no significant interaction between the order in which subjects were tested (test-order) and the condition. The occurrence of a significant test-order effect for the same situation within a condition does not preclude the use of that measure within a condition, but it does preclude its use for making comparisons between the conditions due to the order effect. Likewise, any evidence of a significant episode-order effect for the same situation within a condition precludes the use of that measure from any analysis of episodes occurring at different times within a condition, but does not preclude its use between comparable situations occurring at the same time in the two conditions. However, if both a test-order effect and an episode order-effect are present for the same measure when used in similar situations then that measure cannot be used to make any reliable inferences from the test.

### Proximity/contact seeking

Some of the data relating to ‘proximate owner/stranger’ were found to exhibit an interaction effect (A2 vs B6 (S) z = 108.5, p<0.05), and other data had either a test order effect (B5 vs A1 (O) z = 405.5, p<0.05) or episode order effect (B5 vs A1 (S) z = 419.0, p<0.01) and so this parameter was not used to assess attachment.

Some of the data relating to ‘physical contact owner/stranger’ exhibited a test-order effect (B4 vs A8, z = 108.5, p<0.05) and some an episode order effect (A1 vs B5, z = 266.0, p<0.05) and so this parameter was not considered suitable for use in assessing attachment.

‘Marking’ behaviour exhibited an episode-order effect (A2+A8 vs B6+B4, z = 1107.5, p<0.05; A1 vs B5 [marking owner], z = 263.5, p<0.05; A1 vs B5 [marking stranger], z = 251.0, p<0.01), but no test-order effect and so these data were used in comparisons relating to the same episodes in the two conditions where relevant to the assessment of attachment. There was no significant difference in the amount cats marked the owner versus the stranger in the first episode of test A, but they marked the owner more than the stranger in the first episode of test B. Cats were found to mark the stranger significantly more than the owner when comparing the first separation (A2 vs B2) and first reunion after the cat was alone (A4 vs B4), with no significant difference, when both were present in episode 5 and following the second separation in the next episode(A6 vs B6), but significantly more marking was directed towards the owner than the stranger at the second reunion after the cat had been alone (A8 vs B8) (See Table 3 for statistical results).

**Table 3:** Summary of attachment behaviour results from Wilcoxon signed ranks test. Key: O = owner present, S = stranger present, A1 –A8 = modified condition A episodes 1–8, B1 –B8 = reversed condition B episodes 1–8, Z = Wilcoxon signed ranks test statistic

Behaviour	Episode comparisons	Medians (s)	Test Result
***Proximity/contact seeking***			
Marking	A1(O) vs A1(S)	0 vs 0	Not significant
	B1(O) vs B1(S)	0 vs 0	Z = 125.0, p<0.01
	A2+A8(S) vs B2+B8(O)	6.2 vs 8.3	Z = 171.0, p≤0.001
	A4+6A(O) vs B4+6(S)	40.7 vs 9.7	Z = 0.0, p≤0.001
	A2(S) vs B2(O)	0 vs 1.9	Z = 28.0, p<0.05
	A4(O) vs B4(S)	25.7 vs 5.7	Z = 0.0, p≤0.001
	A5(O) vs A5(S)	6.2 vs 11.1	Not significant
	B5 (O) vs B5(S)	1.2 vs 0	Not significant
	A6(O) vs B6(S)	2.0 vs 0	Not-significant
	A8(S) vs B8(O)	3.3 vs 0	Z = 18.0, p≤0.01
***Secure base effect***			
Exploration/locomotion	A2+A8(S) vs A4+A6(O)	84.1 vs 68.1	Not-significant
	A2(S) vs A6(O)	54.8 vs 27.4	Not-significant
	A4(O) vs A8(S)	20.6 vs 26.0	Not-significant
Passive behaviours	A2+A8(S) vs B2+B8(O)	21.9 vs 0	Not-significant
	A4+A6(O) vs B4+B6(S)	68.1 vs 81.6	Z = 68.5, p≤0.001
	A2(S) vs B2(O)	0 vs 0	Not-significant
	A4(O) vs B4(S)	0 vs 0	Z = 2.0, p≤0.001
	A6(O) vs B6(S)	0 vs 0	Z = 33.5, p<0.05
	A8(S) vs B8(O)	5.9 vs 0	Not-significant
Social play	A2+A8+A1(S) vs A6+A4+A5(O)	0 vs 0	Not-significant
	A2(S) vs A6(O)	0 vs 0	Not-significant
	A4(O) vs A8(S)	0 vs 0	Not-significant
	A1(S) vs A5(O)	0 vs 0	Not-significant
	A1(O+S) vs A2(S)	0 vs 0	Not-significant
	A1(O+S) vs A8(S)	0 vs 0	Not-significant
***Distress when separated***			
Vocalise	A2+A8(O absent) vs A4+A6(S absent)	3.4 vs 4.9	Not-significant
	A2(O absent) vs A6(S absent)	1.7 vs 0.4	Z = 97.0, p<0.01
	A4(S absent) vs A8(O absent)	4.0 vs 0.9	Not-significant

### Secure-base effect

No interaction, test-order or episode-order effects were found for the measures ‘exploration/locomotion’ and ‘social play’ (with the owner or stranger) and therefore these parameters were used further to assess attachment. No significant differences were found in the duration of time cats spent expressing exploration/locomotion in the presence of the owner compared to the stranger (Table 3). Likewise there were no significant differences in the duration of time cats spent playing with their owner compared to playing with the stranger. In addition there was no significant difference in the amount cats played with the stranger when the owner was present (A1) and when the owner was absent (A2 or A8) (Table 3). ‘Passive behaviours’ exhibited episode-order effects (A2 vs B6, z = 368.5, p<0.05; A2+A8 vs B6+B4, z = 023.5, p<0.01) but no test order effects. Therefore this parameter was used to assess attachment only in comparisons of the same episode number between conditions. Cats spent significantly more time expressing passive behaviours in the sole presence of the stranger than the owner in one combination of episodes (A4+A6 vs B4+B6, Z = 68.5, N = 18, p≤0.001), but not the other (A2+A8 vs B2+B8). This significant difference in passive behaviours is evident in both of the individual episode comparisons making up this combination i.e. between A4 and B4 after the cat has been left alone for the first time (Z = 2.0, N = 18, p≤0.001) and between A6 and B6 –the first departure in the second half of the test when (Z = 33.5, N = 18, p<0.05).

### Distress when separated

No interaction, test-order nor episode-order effects were found for ‘vocalising’, therefore analysis focused on condition A, to minimise the risk of Type 1 error. Comparisons within condition A of episodes 2 and 6 showed that cats vocalised more after their owner had departed compared to when the stranger departed (Z = 97.0, N = 18, p<0.01), but there was no significant difference in vocalisations between A4 and A8, which followed the return of the owner or stranger after a period of isolation. When all of these episodes are combined to examine the overall effect of absence of owner versus stranger on vocalisation, there was no significant difference.

Some of the data relating to ‘‘approaching/orientation to the door’ were found to exhibit an interaction effect (A2 v B6, z = 88.0, p<0.05) and so this parameter was not used to assess attachment. The combined data relating to ‘vigilance’ had either a test-order effect (B2 + B8 vs A6 + A4, z = 1463.0, p<0.05) or an episode-order effect (A2+A8 vs B6+B4, z = 1178.5, p<0.05) and so comparisons of vigilance behaviour in the sole presence of the owner versus stranger to assess attachment were not justifiable, nor a comparison between the amount of this behaviour in either of these conditions versus when the cat was alone. There were insufficient data to allow statistical analysis of the parameter relating to contact by the cat with the absent person’s chair.

## Discussion

The aim of this study was to use a fully counterbalanced version of the Ainsworth Strange Situation procedure to explore the extent to which it can be used to infer that the cat-owner bond constitutes a secure attachment [9]. Overall, the response of the cats indicated that the test environment was generally adequate for invoking the typical scenario desired in the ASST for demonstration of a secure base effect. However the specific results indicate that many aspects of the behaviour of cats in this test are not consistent with the characteristics of attachment, for two main reasons. Firstly, relevant aspects of the behaviour of cats are not reliable enough to be used in an evaluation of attachment (i.e. aspects of the test produce unreliable data). Secondly, even among those measures which are temporally robust, the predictions are not met, except in the case of level of vocalisation if it is a proxy of distress, but this alone is not sufficient to imply secure attachment. The additional controls in the current study compared to the previous study [7], which sought to determine whether the cat-owner relationship constituted an attachment as described by Bowlby [8], [9] explains why we reject this hypothesis. Although, we accept it is possible, if unlikely, that the typical relationship between owners and their cats in Mexico is different to that which generally occurs between owners and their cats in the UK., or that there is a difference in the relationship between owners and cats kept indoors (which formed the population tested by Edwards et al., [7]), and cats with outdoor access (which made up the majority of our population). We do not reject that cats may have social preferences, nor that *some* cats might form this type of attachment in certain circumstances, nor do we wish to imply that cats do not form some form of affectionate social relationship or bond with their owners (a broader sense use of the term “attachment”), only that the relationship with the primary caregiver is not *typically* characterised by a preference for that individual based on them providing safety and security to the cat. An alternative explanation for these results might be that the modified SST used here is not an appropriate instrument for measuring attachment, and the finding that the behaviour of cats appears to be very variable (and unreliable across time) may have wider implications for those using behavioural assessments to evaluate cats, such as for rehoming.

In relation to proximity/contact seeking, many measures were found not to be robust enough to be used to evaluate this aspect of attachment. However, the data for marking (in the form of body rubbing), which is a specific behaviour that inevitably results in proximity, were useable in this regard despite showing an episode order effect. The results show there was a shift in the focus of marking within a test. Earlier in the test (episodes 2 & 4), cats marked the stranger more than the owner in comparable situations but as the test progressed there was no preference (episode 6) and finally (episode 8) there was a preference for the owner. This suggests that marking preference *per se* is not indicative of attachment towards the individual being marked, although these results can be explained in another way. It has recently been suggested [39] that marking serves an important function in relation to emotional arousal, with unfamiliar but not overtly threatening objects initially being marked to reduce anxious arousal associated with the uncertainty of the situation, and familiar individuals marked to maintain the social relationship. This allows the efficient allocation of limited attentional capacity. This hypothesis builds on previous suggestions that the purpose of marking between cats is to exchange odours so they become familiarised with one another [23], [29], [40] and laboratory cats have been found to make more direct contacts with an unfamiliar person than with a familiar one [41]. These results are consistent with an expansion of the familiarisation hypothesis that includes a social preference for the owner, as described by Mills et al., [39]. Since the stranger is initially unfamiliar and non-threatening, when the cat is left alone with this individual for the first time (episode 2), due to the departure of the owner, it would be expected that the cat will mark the unfamiliar individual with whom it now finds itself. By contrast in the counterbalanced condition (B), the stranger has just left and the cat finds itself with its owner with whom it is already is familiar. Thus we would predict more marking of the stranger than owner in this episode. Episode 4 represents the first reunion with an individual after the cat has been alone and given the increased familiarity of the owner, the same prediction applies. However, as the test proceeds, the stranger is becoming increasingly familiar to the cat, to the point that by episode 6 the difference that existed in episode 2 is less apparent. Thus in episode 8, which follows the second occasion the cat has been left alone, there is now increased marking of the owner, perhaps because although the two may now both be familiar, the owner is a preferred social contact. Thus the evidence from proximity maintenance/ contact seeking by the cat in support of attachment towards its owner is weak.

The secure-base effect is considered the primary factor in identifying an attachment [10], [42]. However no evidence was found to support the use of owners as a secure-base in the current study. No significant difference was found in the amount of exploration/locomotion in the presence of the owner versus stranger, nor the amount of play with the owner versus stranger. In addition, the absence of the owner did not significantly reduce the time spent playing with the stranger as would be expected if the owner functioned as a secure-base [14]. This might reflect the observation that in cats, unlike humans and dogs, much play is typically associated with solitary predatory type activity, and so may not have a social relevance. Passive behaviours, indicative of relaxation, are suggested as a measure of the secure-base effect in children [10]. However, passive behaviour may not be so easy to interpret in cats [43], [44]. Edwards *et al*., (2007) [7] found that cats were more inactive in the presence of the stranger in their test, and a similar result was found in the current study. Thus it seems that in the context of SST, passive behaviour by domestic cats, may be associated more with a state of anxiety rather than comfort, as has been found in other studies [43], [44]. However, a difference was only found in half of the comparable episodes, and these were the ones in the middle of the test (episodes 4 and 6). If the owner were acting as a secure base within the strange environment, it would be predicted that the effect of their presence over that of the stranger would be greatest at the first separation (episode 2) but this was not the case, since there was no significant difference in the amount of passive behaviour exhibited by the cat at this time. At best it might be argued that the owner has a small effect on the perceived safety of the environment, and this is not strong enough to impact on the behaviour of the cat when it first enters a strange environment, but perhaps as the cat habituates to the environment, the owner’s may have a small effect over that of the stranger. However, this would also indicate that towards the end of the test the cat was sufficiently habituated to the environment, so as not to need the support of another. An alternative and arguably more parsimonious explanation of the finding would relate to the cat’s independence and habituation to the environment and stranger. The strangeness of the environment inhibits the cat (episode 2), and the return of someone after the cat has been alone (episode 4) has a differential effect depending on the identity of that individual. If it is the stranger, then the situation is still novel, whereas if it is the owner it provides a degree of familiarity. The next occasion when a difference is assessed, is episode 6 when either the stranger or owner leaves after they have both been present. The lack of familiarisation with the stranger at this time, would mean the environment is still strange due to their presence, however, by episode 8 (as indicated by the marking behaviour) the stranger has now become familiar and so there is no difference when the cat is left with the owner compared to the stranger. These results examining a potential secure base effect together with those relating to proximity seeking suggest there is a dynamic between the strangeness of the physical environment and the stranger, and that habituation to them occurs at a different rate, occurring to the environment sooner than the stranger, with familiarisation of the stranger being a function of them being marked.

The data relating to potential stress when separated are consistent with a preference for interaction with the owner over the stranger, but not with secure attachment. Standing by the door is a particularly robust measure of separation distress in dogs [13], but was found to be inconsistent in cats, as was vigilance behaviour. This suggests either that cats do not show distress in this way, or that the cats are not particularly distressed by the departure of the owner. By contrast, vocalisation was found to be a robust measure, but differences in vocalisation depending on whether the owner or stranger was absent, are not necessarily consistent with the bond with the owner providing a secure base. Although there was a difference in vocalisation when the cat was left with either the owner or stranger after the other had left (episodes 6 versus 8), there was no difference in vocalisation following the return of the owner or stranger after the cat had been alone. This would be consistent with vocalisation occurring in response to frustration at the owner’s departure, perhaps as a result of previous reinforcement of the interaction (as often occurs at feeding [45]), rather than the owner providing comfort in the strange environment. From a neurobiological perspective separation from a secure attachment figure results in engagement of a different affective system (PANIC *sensu* Panksepp [46] compared to separation from an individual who is associated with physical reinforcements (RAGE *sensu* Panksepp [46]), although both might result in superficially similar behaviour aimed at reinstating contact. In the case of the cat, vocalisation meets the requirement in both situations and so, this measure alone is not sufficient to infer that the cat is attached to its owner as a source of safety and security.

The Ainsworth procedure is suggested to be highly suitable for dogs since it reproduces situations that they are likely to encounter in their everyday lives [14], (potential need for support from their carer). In contrast most cats are unlikely to encounter such situations on a regular basis and it might be argued that this impacts negatively on the validity of the test as it is such an artificial situation. However, in this particular instance and given the theoretical underpinnings of the SST, we suggest that the set-up chosen actually increases the validity of our procedure for the following reasons. The vast majority of cats were home based as is common in the UK and so a novel environment is likely to pose a suitably intimidating challenge to induce the expression of secure attachment related behaviours if they existed [11]. In addition, few of the cats in this study, had much experience of strange situations outside the home. A visit to the vet might be the most likely analogous situation encountered, but none were taken to the vet regularly (for example for treatment of a chronic medical condition); none were reported to be experiencing this type of situation on a regular basis. By contrast, largely outdoor cats who travel a lot to new places, might get used to environmental novelty more readily and this could result in a false negative response. In this regard, it is worth noting that the data from two cat subjects were eliminated from the analysis because their behaviour did not show variation within the test; the hiding and behavioural inhibition observed by these cats are consistent with higher stress levels [43],[44] but it is clear that the presence of the owner was not able to ameliorate these effects, which should be the case if they serve as an attachment figure in the original sense of Bowlby [8], as compared to the wider sense used by some authors [47].

It might be argued that, the behaviours chosen to assess attachment are not biologically relevant given the nature of the cat as a largely independent, solitary hunter. However, this aspect of the cat may be precisely the reason why the relationship with the owner is not characterised by the safety and security features of a classical attachment bond. Even when accounting for a different function in superficially similar behaviour categories between species (such as passiveness), the evidence from the current study refutes the notion that cats normally show attachment to the owner in the way Bowlby defined attachment [8] and has been found in dogs [13] and claimed to occur in cats [7]. Despite this, there is good evidence that some cats can show separation related problems [6] and there are several possible reasons for this. It may be that a sub-population of cats showing clinical signs do become genuinely attached in the way described by Bowlby, but we consider this unlikely if attachment has a strong biological function and in light of some of our unpublished observations. An alternative explanation is that these problems are perhaps more of a response to frustration at owner absence [39]. This hypothesis lays the foundation for further research and the development of more specific intervention protocols as a result.

Although cats can be social, sociality is likely to exist on a continuum, varying between individuals, but perhaps skewed towards independency. They have been domesticated for a relatively short time in comparison to dogs and have not been selectively bred to live in close contact with people [27], nor is their natural social system highly dependent on the same type of close social bonds [23]. Indeed, within the human-cat relationship the frequency and duration of interactions have been observed to be low in comparison to dogs [35]. These factors are likely to affect the nature of the relationship that typically forms between cat and owner, and make the formation of cat-human attachment unlikely. Nonetheless, some may be capable of forming very strong attachments, but this would not seem to be the norm. However, cats do seem to have a preference for their owner over an unfamiliar individual but the extent to which this is conditioned or the result of an intrinsic psychological tendency to bond remains unclear.

## Conclusion

It seems that generally cats do not appear to attach to owners as a focus of safety and security in the same way that dogs do or children do towards their parents as demonstrated by their behaviour in a strange situation test. However, cats do appear to have a different relationship with their owner compared to a stranger, but the extent to which this is conditioned as a result of incidental interactions or built upon the fulfilment of an intrinsic psychological social need is unknown.

## Supporting Information

S1 File List of Behaviours. (PDF)

S2 File Cross-over design. (PDF)

S3 File Cross-over design analysis results using Mann-Whitney. (PDF)
