# Supplementary material for: Domestic Cats (Felis silvestris catus) Do Not Show Signs of Secure Attachment to Their Owners
Source: PLoS One. 2015 Sep 2;10(9):e0135109. doi: 10.1371/journal.pone.0135109 (PMC4558093; doi:10.1371/journal.pone.0135109)
Supplement: S1 File — (PDF) [file pone.0135109.s001.pdf]

Supporting Information
S1 List of Behaviours

Behaviour	Description
Passive exploration:	Gathering of information from the environment from a stationary position, by visually scanning the area and/or by sniffing. The cat may do this from a standing, sitting or lying position [39].
Active exploration:	Gathering of information from the environment whilst moving around at a walk or trot, by sniffing, visual inspection and oral examination. Cat may stop intermittently to further investigate a particular area [30].
Locomotion:	Walking, pacing or running around without exploring the environment or playing [13].
Play involving another:	Any manipulating, patting, throwing, pouncing, chasing, wrestling of toys involving the active interaction with the owner or stranger.
Independent play:	Manipulates an object with its paws in an apparently playful manner, without active engagement of another. Cat may also chase or paw at its own tail [40].
Approaching/orientation to person:	Includes cat moving towards the owner or stranger, sitting, standing or lying down in the vicinity (0.3 m) of the owner or stranger.
Marking:	Rubbing face or body against an object or person [7].
Physical contact:	Any physical contact with the owner or stranger initiated by the cat excluding marking behaviour.
Following:	Following the person around the room or to the door [12].
Approaching/contact with the door:	Includes cat approximately the door, touching it, lying down or staying still in the vicinity of the door (0.3 m) [7].
Vigilance:	In a state of alertness. Includes cat sitting, standing or lying down with eyes fully open, flicks ears occasionally as it scans its surroundings [40].
Passive behaviours:	Standing, sitting or lying down without any obvious orientation towards the physical or social environment [12].
Vocalisation:	Includes meow, chirrup, trill, growl, yowl, hiss, purr, spit [40].
Contact with owners/strangers empty chair:	Cat sits, stands, lies down on or stands up on hind legs with forelegs on owners/strangers empty chair.
Self-grooming:	Cat grooms itself by licking its body or by licking its paw and passing over its head. Grooming can include scratching and brief chewing of the body [40].
Hiding:	Cat is located in hiding space provided in either testing room (Figure 1)
