# Supplementary material for: Domestic Cats (Felis silvestris catus) Do Not Show Signs of Secure Attachment to Their Owners
Source: PLoS One. 2015 Sep 2;10(9):e0135109. doi: 10.1371/journal.pone.0135109 (PMC4558093; doi:10.1371/journal.pone.0135109)
Supplement: S2 File — (PDF) [file pone.0135109.s002.pdf]

Supporting Information

S2 Cross-over design

Behaviour	Episodes
Proximity/contact seeking	
Following and approach	A2 and A8 and<br>B6 and B4(S)<br>B2 and B8 and<br>A6 and A4(O)<br>A2 and B6 (S)<br>B2 and A6 (O)<br>A4 and B8 (O)<br>B4 and A8 (S)<br>A1 and B5 (O+S)<br>B5 and A1 (O+S)
Physical contact	A2 and A8 and<br>B6 and B4(S)<br>B2 and B8 and<br>A6 and A4(O)<br>A2 and B6 (S)<br>B2 and A6 (O)<br>A4 and B8 (O)<br>B4 and A8 (S)<br>A1 and B5 (O+S)<br>B5 and A1 (O+S)
Marking	A2 and A8 and<br>B6 and B4(S)<br>B2 and B8 and<br>A6 and A4(O)<br>A2 and B6 (S)<br>B2 and A6 (O)<br>A4 and B8 (O)<br>B4 and A8 (S)<br>A1 and B5 (O+S)<br>B5 and A1 (O+S)
Secure-base effect	
Exploration/locomotion	A2 and B6 (S)<br>B2 and A6 (O)<br>A4 and B8 (O)<br>B4 and A8 (S)<br>A2 and A8 and<br>B6 and B4(S)<br>B2 and 8B and<br>A6 and A4(O)
Passive	A2 and B6 (S)<br>B2 and A6 (O)

	A4 and B8 (O)
	B4 and A8 (S)
	A2 and A8 and B6 and B4(S)
	B2 and B8 and A6 and A4(O)
Social play	A2 and A8 and B6 and B4(S)
	B2 and B8 and A6 and A4(O)
	A2 and B6 (S)
	B2 and A6 (O)
	A4 and B8 (O)
	B4 and A8 (S)
	A1 and B5 (S)
	B1 and A5 (O)
Distress when separated	
Vocalising	A2 and A8 and B6 and B4(S)
	B2 and B8 and A6 and A4(O)
	A2 and B6 (S)
	B2 and A6 (O)
	A4 and B8 (O)
	B4 and A8 (S)
Approaching door	A2 and A8 and B6 and B4(S)
	B2 and B8 and A6 and A4(O)
	A2 and B6 (S)
	B2 and A6 (O)
	A4 and B8 (O)
	B4 and A8 (S)
	A3 and B7 (C)
	A7 and B3 (C)
Vigilance	A2 and A8 and B6 and B4(S)
	B2 and B8 and A6 and A4(O)
	A2 and B6 (S)
	B2 and A6 (O)
	A4 and B8 (O)
	B4 and A8 (S)
	A3 and B7 (C)
	A7 and B3 (C)
Contact chair O/S	A2 and B6 (S)
	B2 and A6 (O)
	A4 and B8 (O)
	B4 and A8 (S)
	A3 and B7 (C)
	A7 and B3 (C)
