# Supplementary material for: Domestic Cats (Felis silvestris catus) Do Not Show Signs of Secure Attachment to Their Owners
Source: PLoS One. 2015 Sep 2;10(9):e0135109. doi: 10.1371/journal.pone.0135109 (PMC4558093; doi:10.1371/journal.pone.0135109)
Supplement: S3 File — (PDF) [file pone.0135109.s003.pdf]

Supporting Information

S3 Cross-over design analysis results using Mann-Whitney.

Behaviour	Episodes	Interaction	Test order effect	Episode order effect
Proximity/contact seeking				
Proximate owner/stranger	A2 vs A8 vs B6 vs B4(S)	X	X	X
	B2 vs B8 vs A6 vs A4(O)	X	X	X
	A2 vs B6 (S)	✓ Z = 108.5*	NA	NA
	B2 vs A6 (O)	X	X	X
	A4 vs B8 (O)	X	X	X
	B4 vs A8 (S)	X	X	X
	A1 vs B5 (O)	X	X	X
	A1 vs B5 (S)	X	X	X
	B5 vs A1 (O)	X	✓ Z = 405.5*	X
	B5 vs A1 (S)	X	X	✓ Z = 419.0**
Physical contact	A2 vs A8 vs B6 vs B4(S)	X	✓ Z = 1496.5**	X
	B2 vs B8 vs A6 vs A4(O)	X	X	X
	A2 vs B6 (S)	X	X	X
	B2 vs A6 (O)	X	X	X
	A4 vs B8 (O)	X	X	X
	B4 vs A8 (S)	X	✓ Z = 386.5**	X
	A1 vs B5 (O)	X	X	✓ Z = 266.0*
	A1 vs B5 (S)	X	X	X
	B5 vs A1 (O)	X	X	X
	B5 vs A1 (s)	X	X	X
Marking	A2 vs A8 vs B6 vs B4(S)	X	X	✓ Z = 1107.5**
	B2 vs B8 vs A6 vs A4(O)	X	X	X
	A2 vs B6 (S)	X	X	X
	B2 vs A6 (O)	X	X	X
	A4 vs B8 (O)	X	X	X
	B4 vs A8 (S)	X	X	X
	A1 vs B5 (O)	X	X	✓ Z = 263.5*
	A1 vs B5 (S)	X	X	✓ Z = 251.0***
	B5 vs A1 (O)	X	X	X
	B5 vs A1 (S)	X	X	X
Secure base effect				
Exploration/ locomotion	A2 vs B6 (S)	X	X	X
	B2 vs A6 (O)	X	X	X
	A4 vs B8 (O)	X	X	X
	B4 vs A8 (S)	X	X	X
	A2 vs A8 vs B6 vs B4(S)	X	X	X
	B2 vs B8 vs A6 vs A4(O)	X	X	X
Passive behaviours	A2 vs B6 (S)	X	X	✓ Z = 368.5*
	B2 vs A6 (O)	X	X	X
	A4 vs B8 (O)	X	X	X
	B4 vs A8 (S)	X	X	X
	A2 vs A8 vs B6 vs B4(S)	X	X	✓ Z = 1023.5***
	B2 vs B8 vs A6 vs A4(O)	X	X	
Social play	B2 vs B8 vs A6 vs A4(O)	X	X	X
	A2 vs A8 vs B6 vs B4(S)	X	X	X

	A2 vs B6 (S)	X	X	X
	B2 vs A6 (O)	X	X	X
	A4 vs B8 (O)	X	X	X
	B4 vs A8 (S)	X	X	X
	A1 vs B5 (S)	X	X	X
	B1 vs A5 (O)	X	X	X
Distress when separated				
Vocalising	A2 vs A8 vs B6 vs A4 (S)	X	X	X
	B2 vs B8 vs A6 vs A4(O)	X	X	X
	A2 vs B6 (S)	X	X	X
	B2 vs A6 (O)	X	X	X
	A4 vs B8 (O)	X	X	X
	B4 vs A8 (S)	X	X	X
Orientating/approaching door	A2 vs A8 vs B6 vs A4 (S)	X	✓ Z = 1118.5*	X
	B2 vs B8 vs A6 vs A4(O)	X	✓ Z = 1149.0*	X
	A2 vs B6 (S)	✓ Z = 88.0*	NA	NA
	B2 vs A6 (O)	X	X	X
	A4 vs B8 (O)	X	X	X
	B4 vs A8 (S)	X	X	X
	A3 vs B7 (C)	X	X	X
	A7 vs B3 (C)	X	X	X
Vigilance	A2 vs A8 vs B6 vs A4 (S)	X	X	✓ Z = 1178.5*
	B2 vs B8 vs A6 vs A4(O)	X	✓ Z = 1463.0**	X
	A2 vs B6 (S)	X	X	X
	B2 vs A6 (O)	X	X	X
	A4 vs B8 (O)	X	X	X
	B4 vs A8 (S)	X	X	X
	A3 vs B7 (C)	X	X	X
	A7 vs B3 (C)	X	X	x
Contact with O/S empty chair	A2 vs B6 (S)			
	B2 vs A6 (O)			
	A4 vs B8 (O)		Insufficient data	
	B4 vs A8 (S)			
	A3 vs B7 (C)			
	A7 vs B3 (C)			

Key:

(S) = Stranger present, (O) = Owner present, (C) = Cat alone,

X = no significant difference , ✓ = significant difference, * = p<0.05, ** = p<0.1, *** = p<0.001

Z = Mann-Whitney statistic test number
